# Editorial and Miscellaneous

**Published:** 1862-08

**Authors:** 


CHICAGO

MEDICAL JOURNAL.

VOL. V.] AUGUST, 1862. [No. 8.

### EDITORIAL AND MISCELLANEOU

*The Domain of Medical Police.* (Abstract of a paper read before the N. Y. Sanitary Association, Feb. 6th, 1862.) By LOUIS ELSBERG, M. A., M. D. Pamphlet, pp. 16.

Slowly, but steadily and surely, the thinking men of the profession are advancing toward the clear apprehension of the great truth that the larger part of the true physician's office consists, not in the administration of drugs, whether as prophylactics, specifics or "even to meet the indications," but in the discovery and control of those other and larger influences which beget and continue disease.

Disease is beginning to be understood as an orderly phenomenon—it has its laws as assuredly as health. Conditions change and results are changed. The normal order of relations of the organs and particles of the body to things without and within being modified, we have pathology in place of physiology—but all the time *order*.

Our duty as physicians is to bring back the normal order by controlling all disturbing causes. This is simple enough, but it is not exactly clear why a case of Typhus, begotten and sustained by bad air, food, water, fatigue, etc., etc., should engender, not especial attention to these things, but to Emetics, Verat. Virid, Turpentine and Chlorate of Potash. Certain adroit combinations of these and others are thought better adapted to restore to health than taking an immense amount of time and trouble to find out and remove atmospheric and terraqeous disturbing agents. It is easier to feel pulses and look at tongues, and then write a prescription or dole out a parcel of pills. It looks wiser to name the disease on the instant and write its remedy in a *billet doux* to the druggist the next. A method compendious and convenient enough, but unfortunately altogether fallacious.

Dr. Elsberg, in the pamphlet before us, does not undertake to elucidate the whole subject, but in his own words: "to run over the volume as if it were before us, *not* in its separate pages, but by its preface and table of contents." And we notice his essay not to reproduce its contents or even to give a systematic outline of its topics, but merely to detach a sentence here and there, and heighten attention to the general subject matter.

After defining the term police, and explaining its necessity to civil society and adverting to the limits of its compulsory power, particularly where its duties touch upon the general health, remarking that medicine bears to police, as medical police, a similar relation to that existing between it and law as medical jurisprudence—he continues:

The *domain* of medical police admits of a division into three departments: (1.) Preservation of Public Health; (2.) Removal of Disease; (3.) Administration of Medical Affairs. These three divisions link, at many points, however, closely into each other. The first embraces not only the maintenance, but also the promotion of improvement of the existing sanitary condition of the community, primarily therefore the exclusion of the causes of disease; the second includes the re-establishment of the perturbed state of public health, specially therefore the cure of diseases; while the third embraces the totality of the various laws, regulations, and institutions referring to medicine.

Medical police should be in the hands of competent medical men. It is true all medical men may not be competent to the position, but certainly none but a medical man is. To secure a competent medical *personnel*, the State should provide for and regulate medical education and practice. It should regulate the supply and sale of drugs, medicines, poisons, etc., etc. During the prevalence of epidemics, the duties of the medical police are especially important. It should build and sustain hospitals; prevent premature interments, and provide for rescue from apparent death, drowning, etc. Accidents of all sorts should be within its purview, both for immediate assistance and prevention of repetition.

*Par excellence*—it should act as a *Sanitary* Police.

The most important and fertile source of *effective public hygiene* is thorough, minute, and extensive SCIENTIFIC OBSERVATION of the *sanitary condition* of the community, and the *causes* influencing it. That such observations, or in other words, *skilled sanitary inspections*, can be made only by persons of a medical education—that private physicians cannot make them extensively and continuedly enough for the purpose, and that they require specially appointed *health officers*, surely is too evident to every intelligent person to require much elucidation.

Let any one but *consider* (even if ever so superficially) what duties such inspection involves. It must embrace the whole manner—the *tout-ensemble*—of a man's life and his relation to all naturall and artificial agencies affecting health. It must yield scientific STATISTICAL, CHEMICAL, MICROSCOPICAL and TECHNOLOGICAL results for sanitary purposes. It requires a health officer to prepare, and keep corrected, a SANITARY MAP, (of the district alloted to his inspection,) showing the grade, width and condition of the streets, the sewers, the soil, the vacant lots and buildings, and a SANITARY REGISTER, showing the number and condition of the inhabitants; not only the deaths, but also the diseases occurring in each locality, with meteorological, barometric and thermometric records, as well as occasional chemical and microscopical analyses of the air and the drinking water of a particular locality, of particular articles of food, etc. I do not mean to say that every *medical man* is fit for such inspection, but that none except a person of a *medical education* IS! A health officer, besides making the ordinary medical studies, must pay *particular*

*attention* to the causation, eradication and prevention of disease. And here, allow me for a moment to enter into an explanation of the *term* "disease." Although it is convenient in description and discussion to speak of disease as an entity, all educated physicians (if not, indeed, all educated persons,) are well aware that what is so designated, has really *no* personal or ontological character, but is only a condition, and may be defined "a *perversion* either of the functions or of the structure of the body, or of any of its parts." It is, in other words, "a deviation from the normal physiological state or action of the organism under the disturbing influences of morbid causes." In this respect, the positive progress of physical science enables us to stand to-day on altogether a different footing from that of our forefathers. "All pathology is but the physiology of organic perturbations."* The organism never becomes diseased by itself, nor does it ever alone become a cause of disease. Being the source of all manifestations of life, it is at the same time, also, the means of preservation of these manifestations; only that in its material constitution lies the general possibility of becoming diseased. But the realization of this possibility requires the addition of another factor, that may be called morbific—or literally, sick-making—Cause!

The morbific cause must, however, not be regarded a unit. In all cases there are really two different factors, only the co-action of which makes up a cause: producing disease as such —*i. e.*, that calls forth the phenomena giving rise to the term. But in many cases, the organism is forced, by original or acquired constitution, to act as second factor when one factor of a morbific cause has gone into operation. These factors are called, respectively, remote or predisposary, and direct or exciting cause. (One alone has also been called the cause, and the other the totality of circumstances under which it can act.)

Every man, even the healthiest, as we find him, has natural predispositions to disease; every one will therefore, under favoring circumstances, become sick. Predispositions as well as morbific causes are very numerous and diverse. They are all of interest and importance to the practicing physician, but medical police is specially concerned with such of them only as transcend the ability of the individual to exclude. As to

* Memoranda Medica, or Note-Book of Medical Principles. By Henry Hartshorne, A. M, M. D. Philadelphia, 1860.

others than such, its province can only extend, according to circumstances, to calling attention, by appropriate instruction and warning to the danger and the means of avoiding it; as to the former, however, it has the right and the duty to enforce regulations for their exclusion.

The branch of medicine specially concerned with the *annihilation of the causes of disease* has, during the last few years, found many ardent investigators! A *new name: Nosophthory*—literally, the science of eradicating disease—has been given to it, and it bids fair to attain the rank of honor among the medical sciences!

The SURVEY of this domain of Sanitary Police (i. e., *the exclusion of morbific causes,*) may, for our present purpose, be divided into three sections, viz: I. Total Extinction of Causes of Disease. II. Protective Measures against Contagious Diseases. III. Protective Measures against Miasmatic Diseases.

I. The total eradication of certain diseases may be considered under the two sub-heads: A. Prevention of hereditary diseases. B. Removal of injurious external influences.

We have given this long extract because of its sound truth, and because it sustains the views which for many years we have labored to inculcate to medical students, and diffuse among medical men.

Civilization has been too long looked upon as necessarily introducing elements of disease and abbreviation of life unknown to man in the so-called state of nature. But the truth is, a true civilization lengthens indefinitely the average term of life. Appropriate understanding of them will by and by not merely render the causes of disease innocuous, but, moreover, render them subservient to still higher development of man's material and mental organization.

Under the heads given Dr. E. glances at Hereditary Diseases; Illegitimate Procreation; Injurious External Influences; Foundling Hospitals; Lying-in Asylums; Physical and Mental Education of Children; Food and Drink and their Adulterations; Articles of Manufacture; Domiciles; Streets, Docks, &c.; Public Buildings; Removal of Injurious Effects of Trades, &c.; Burial Grounds; Sale of Poisons;

Precautions against suicide, drunkenness, injuries from the insane, and various accidents, obstructions, excavations, explosions, fires, &c.; Protective measures against Contagious and Miasmatic diseases; Epidemics, Endemics, Typhus, &c., &c. Then:

In conclusion, let me repeat five words, which we can never too often reiterate in the ears of the public and of our Legislature—five words of *Truth!* and *Hope!!* and *Charity!!!*

"*All malignant epidemics are preventable.*" "By attention to the laws of public and domestic hygiene, all malignant epidemics are preventable." This truth must become the gospel text of the Evangelists of Sanitary Reform! Promulgate through all the avenues to the public mind, every civilized community must appreciate its import, and would soon, by appropriate sanitary measures, realize some of its promises!

"All malignant epidemics are preventable," AND CAN BE PREVENTED by attention to the laws of hygiene, which it is the object of *this Association* to have systematized and acted upon in the Metropolitan Health District! SHALL *they ever be prevented among us?*

Well did the Sanitary Committee of our Board of Health for 1849, say: "To no other work should the authorities address themselves more earnestly than the establishment of a *thoroughly organized medical police.* * * * The *advantages* of such a measure would be *incalculable.*"

Well did the State Senate Select Committee, in 1859, say: "Preventive Medicine, rising rapidly to the perfection of a science, is capable of exerting a vast influence over the welfare, physical and moral, of the human race!"

Well did Dr. Griscom, *before this Association*, but a few months ago, quote Mr. Simon's words: "*Preventive Medicine* will effect INFINITELY MORE for mankind than all the drugs which have *yet been discoved*, and all the *curative skill* which has *ever* been exerted for the *alleviation of disease.*"

If ever any city could profit by the establishment of a medical police, with the enlarged scope Dr. Elsberg has indicated, this city of Chicago most certainly is one which would; but the experiment, if it may be so termed, has yet to be tried.

This city illustrates on the large scale the salutary effects of free ventilation. Its ventilation is the only thing which prevents it from being a Northern Cairo (we do not refer to Cairo

Illinois, but to its *old* Egyptian namesake.) The site is low and flat, abounding in surface water, stagnant pools and reeking vegetable decomposition. Art has lifted quite a portion of the central city a few feet above the perpetual surface water, and a certain amount of tardy sewerage is possible. But much of the inhabited part of the city is still upon the old level, with water standing in the cellars, and rank with dead dogs, dead cats, offal and garbage of every description in the alleys and back streets, which need but trifling excavations to be canals. The sewers and drains nominally find exit into the river from much of the general surface, but as the river has no fall, and fluid will not rise higher than its source, the only effect is conversion of its contents into a "gruel thick and slab" black as Erebus, yet its surface in the sunlight prismatically colored from creaming grease, and sending heavenward constantly odors more pungent and foul than the nine-and-thirty stinks of Cologne. The hog and cattle yards and slaughter houses, the distilleries and breweries, and worse than all a hundred fold, the gas-houses discharge their accumulated abominations into the same reservoir, and the commingled "stench thereof ascends to heaven!"

In the palatial residences of the wealthy, and in the cellars of the poor, everything is damp—every material object, as Signor Mantalini remarked, "is a dem'd, moist, unpleasant body." Gas pipes leak in all possible places. The festering garbage and offal of the graded streets and alleys are every now and then gathered into piles and carted off to assist in filling up those "below the grade," and the sun and the rains mingle the decomposing ordure into a compost vile beyond the delineating faculty of a Dickinson to depict and vituperate.

And yet to-day Chicago is habitable, indeed pleasantly so, and what is more, no healthier city is to be found on the continent. "There is a Divinity which shapes *its* ends," and allows the wind to blow where it listeth untaxed, and unarrested by the police. Fortunately for us the "Board," whatever its other powers, cannot control the winds or the weather. The lake shore, and the vast unoccupied spaces in the city limits, held by speculators and waiters upon Providence, afford breathing spaces and ventilating draughts. Thus the winds, which strike the stranger to Chicago so rawly and disagreeably, are our salvation, whilst of human "medical police" there are none or worse than none.

We venture the assertion that the co-operation of an enlightened board of medical police, with a comparatively trivial pecuniary expense, in a single season would render Chicago a marvel of salubrity.

Meanwhile we are at the mercy of the winds, and of a "Board" and "Council," who are also sufficiently windy and who fail to discover facts immediately under their corporate nose. We expect when the war is over, and men have time to reflect that preserving life is more noble than destroying it—then we may have a medical police. Before the war lives were too plenty already, and the disciple of Malthus in better vogue than the Æsculapian. Perhaps after it, there may be felt a public necessity for a new stock of vitality, and methods of preservation be adopted which will illustrate the general belief that the life of a man is part of the wealth of the State, and that his health is not only an individual but a public advantage.

At the present not even the medical officers of the army can get respectable attention paid to the subject—in civil life the effort is altogether Utopian.

---

*On Military and Camp Hospitals and the Health of Troops in the Field.*—Being the results of a commission to inspect the sanitary arrangements of the French Army and incidentally of other armies in the Crimean War. By L. Bauden,s Inspector and Member of the Council of Health of the French Armies, etc., etc. Translated and Annotated by Franklin B. Hough, M. D., late an Inspector of the U. S. Sanitary Commission. New York: Bailliere Brothers, 440 Broadway; 1862. From the Publishers.

This is a very readable and interesting little volume of 260

pages, prepared by the author at the request of Marshal Vaillant, the French Minister of War. It is especially noteworthy from the important practical suggestions it contains with regard to the hygiene of camps, hospitals, etc. The most approved French methods are explained, and frequent comparisons with those of the English instituted, so that each may profit by the experience of the other. Incidentally, a considerable amount of surgical information is communicated.

Among the subjects discussed we note the medical topography of the Crimea; rations; camps and shelters; clothing; field hospital and medical service; surgical operations; chloroform; cholera; typhus in the Crimea; Scurvy; the Constantinople Hospitals, etc., etc. The translator appears to have performed his part of the duty carefully and judiciously.

---

*The American Journal of Ophthalmology, July*, 1862.—JULIUS HOMBERGER, M. D., Editor and Proprietor. Published by Bailliere Brothers, 440 Broadway. 48 pages monthly. $2.00 *per annum.*

It is a bold undertaking to start a new medical journal in these times of wars, fightings and financial depression; and it would seem especially so if that journal is to be devoted to a specialty. Nevertheless, we trust the American Journal of Ophthalmology will prove a success. It is well printed; the contents of importance and interest, and the editor thoroughly competent for the position. We trust he will succeed in redeeming this specialty from the "class of mercenary, irresponsible and unconscientious adventurers" to which its practice is now almost given over. There is no fear that any specialty will be studied too much or elucidated too fully—the danger is quite otherwise than that.

---

*Caries of Elbow Joint;* Operation of Excision with Recovery of Useful Arm. Presented to the New York State Medical Society at its annual meeting, 1862. By N. C. HUSTED, M. D., New York City. Pamphlet 16 pp. From the author.

Aside from the details of the particular case successfully treated by excision, Dr. Husted has compiled a brief history of the operation based mainly on the monograph by Dr. R. M. Hodges, of Boston. He endorses fully the following conclusions by Dr. Hodges:

First.—That although partial excision had been practiced upon several occasions, and by Mr. Wainman in England, so early as 1758-59, the first complete excision of the elbow joint was performed by the elder Moreau in 1794.

Second.—That excision for traumatic cause appears to be a safer operation than amputation, and ordinarily preserves a limb of very considerable usefulness. This is especially true of those occurring in civil practice.

Third.—That excision for anchylosis is only adapted to cases where the arm has stiffened in a straight position, or in one of extreme flexion, unless special circumstances authorize the risk of an operation frequently ending in no improvement of the anchylosis.

Fourth.—That in excision for disease death occurs once in 7 14 15 cases; and that the operation fails of its primary intention—the riddance of the disease with the preservation of a useful arm—by death, amputation or other cause, once in 3 17-34 cases. Patients recovering are usually able to resume their ordinary occupations.

Fifth.—That partial excision, either for traumatic or organic lesions, is a frequent cause of unfavorable results."

---

*Fungi of Wheat Straw the Probable Source of "Camp Measles," and perhaps of Measles Generally.*—Dr. J. H. Salisbury, of Newark, Ohio, in a communication to the *Amer. Journal of Medical Science*, propounds the idea that the fungi of wheat straw are the probable source of the measles which has proved the pest of the camps in both armies, and perhaps of measles generally. The fungi, he remarks, belong to the lowest types of vegetable existence, but, unlike flowering plants, they absorb oxygen and exhale carbonic acid. Decay is an essential condition to their development. In their chemical constitution the large per centage of azotized matter they contain likens them more to animals than to plants. Most of them have but a few hours of growth and maturity, and then as rapid a decay. Their characteristics vary with their peculiar nidus. Even when appearing on living tissues they evidence decay of particles—thus even animals may present parasitic fungi capable, often, of being communicated from one individual to another. In man *porrigo favosa*, *sycosis menti*, and *aphthæ* are examples. Their individual cells and spores being microscopic, their detection is usually difficult and, we may readily conceive, sometimes impossible. Yet, undoubtedly, they sometimes produce deleterious effects by their presence alone, no further development occurring.

Hon. J. Dille, of Newark, called the attention of Dr. S. to the matter, he attributing an attack of sickness, accompanied with the usual symptoms of measles in a tolerably severe form, to inhalation of the dust from an old straw stack, portions of which were partially decayed. He could taste the old straw in his throat for days. The coryza and eruption were distinct together with soreness of the throat pain, delirium and fever.

At Camp Sherman, in the same place, strawticks in a state of decay were coincident to the prostration of a large number of the men (some forty or fifty at once) with measles. The most careful inquiry failed to ascertain any source from which it could have been imported, but individuals exposed to the first cases subsequently took the disease. Several other cases are adduced; and it is also stated that the same effects had attracted the attention of the Farmer's Club, and it is also noticed that swine growers who bed their hogs with straw are very liable to similar symptoms.

After a very careful microscopic examination of the various fungi affecting the straw, the forms of which he has given in the *Journal*, he proceeded to inoculate himself, and afterwards his wife and eleven others, with the result in from twenty-four to seventy-two hours of lassitude, chilliness, catarrhal symptoms, pains through the forehead and temples, and sometimes blotches on the surface. "The disease commenced in the head, throat and fauces, and passed downward, the bowels being very sore after the head, throat and chest were relieved."

The disease produced by inhalation of the spores or by inoculation, manifests the eruption in from twenty-four to ninety-six hours.

Dr. S. calls upon the profession to make observations and experiments upon this subject and report progress. He observes: "To what extent inoculation with straw fungi may prove effectual in protecting the human system against the contagion of measles, can only be settled by careful and extended experiments."

The subject is an important and interesting one, and although the observations and experiments made by Dr. S. are not sufficient to warrant the formation of an opinion, yet it can do no harm, and may do good to follow up the hint he has afforded.

---

*Small Pox.*—In the same journal, Prof. S. H. Dickson has an article urging further provisions to secure perfect vaccination. He would have strict police regulation of the subject making vaccination obligatory upon all. After arguing at some length, he says: "Let us procure that it shall be ordained that every child shall undergo vaccination by some expert within a month after birth; that as soon as the constitution shall have gone through its influence, inoculation with variolous virus shall be performed, and that this latter operation shall be repeated again and again at brief intervals, until all reasonable satisfaction has been attained of the entire extinction of the susceptibility to small pox."

The careless and inefficient manner in which vaccination is too generally performed—the use not only of improper materials, but of uncertain methods, and the failure to repeat it at suitable intervals, have gone far to lessen confidence in the process itself. Proper attention being paid to these points, we believe the protective power of vaccinia fully equal to that of variola itself, without the dangerous exposure of third parties incident to the process of innoculation.

---

*Burns.*—Dr. John Ashurst, Jr., in the same journal, discusses burns. He argues the necessity of prompt and judicious treatment. At first the burn is to be looked upon more as a constitutional than as a local affection. Put the patient to bed, wrap him up in blankets and administer say sixty drops of laudanum and an ounce of brandy. The shock of extensive burns is the most to be dreaded, and brandy and opium are the cardinal remedies. The amount which will be borne is surprising. If the patient is drunk at the outset, give ammonia freely. Dr. A. has given for weeks and weeks a pint a day of brandy in the form of milk-punch, and half a grain of morphine every six hours, and this was no more than enough to quiet the nervous jactitation and restlessness.

If half of the surface is involved in the burn, no matter how superficial, the patient will almost surely die. Even one third, if it be over the trunk, will almost necessarily prove fatal. Burns are the most mortal and *most* deceptive injuries the surgeon is called upon to treat. Dress a small portion first, say the arms, then the trunk and legs, then the face. Dr. A. generally employs the old "carron oil"—linseed oil and lime-water. The zinc paint, Kentish's ointment, solution of lunar caustic, are inferior in his opinion.

The best method, he thinks, is to soak pieces, not more than eight inches square, of patent lint, Canton flannel, or old linen or cotton in the mixture, apply accurately over the parts and cover the whole with oiled silk. He prefers these to the carded cotton. The dressings should be retained in place by roller bandages. The patient should be encouraged to make hearty meals. The state of the bowels and urinary organs should be carefully attended to. Great care should be exercised that he does not drink too much water. Bits of ice, carbonic acid, water, etc., may be employed, but never over a mouthful of fluid at once.

If the reaction is violent and accompanied with delirium, it requires the treatment of mania-a-potu—brandy, opium, lupulin, valerian and the category of nerve stimulants. The sores should be dressed *as seldom as possible*, and after the sloughs have separated, simple cerete, and subsequently carb. zinc cerate or ung. zinc oxid. may be substituted for the carron oil on the patent lint.

Great care should be exercised in cleansing and dressing the sores. Cold should be excluded and the water used rather above than below the temperature of the room. The raw surface should not be touched by the sponge, but the surrounding skin should be carefully sponged. When granulation and cicatization begin it is advantageous to squeeze from a sponge a little whiskey over the surface. This causes considerable pain, but if the dressing is speedily applied it is only momentary. Occasionally the edges need touching with lunar caustic in substance, and constriction is to be prevented by suitable splints and bandages. He cannot agree with Keutish in recommending low diet and purgatives during the suppurating and granulating stage, but urges full diet, tonics and stimulants, especially Tr. Ferri Muriat, Quinia and Acid. Sulph.

Coma, which sometimes occurs in thé early stage, forbids the use of opium. Tetanus is to be combated by Cannabis Indica. This remedy he has given with success in doses of one grain of the extract every two hours.

He is convinced that careful observance of these rules, which have been impressed upon his mind by sad experience, would vastly lessen the mortality of burns, so long considered the opprobrium of surgery. Briefly, the general condition is *first* to be attended to. A burn is essentially a disease of depression, not of excitement. There is nothing to be eliminated—no "fire to be drawn out." None to be given up, until *in articulo mortis.* Immediate action is demanded, and hesitancy and vacillation are positively criminal.

We are a little surprised that the author does not allude to the local application of flour in these cases. It is always readily to be procured, and we have seen striking relief from its use in seemingly desperate cases.

*Traction of India Rubber in Fractures of the Femur.*—JOHN H. PACKARD, M. D., commends the use of India rubber cords or straps for the purpose of keeping up the steady and unintermitting traction needed in fractures of the thigh, the idea having been suggested to him by the elastic "accumulator," commended by Barwell in his "Dis. of the Joints." A thick and strong piece of rubber should be used, as continuous stretching impairs the elasticity of small pieces. He figures his own apparatus, but methods of application will readily suggest themselves to every practitioner.

---

*Paraplegia.*—In a recent clinical lecture, T. GAILLARD THOMAS, M. D., Physician to Bellevue Hospital, tabulates the comparison between organic and functional paraplegia. We reproduce from the American Medical Monthly:

| *Organic Paraglegia.* | *Functional Paraplegia.* |
|---|---|
| 1. Comes on suddenly, and is more or less complete. | 1. Comes on gradually, and is always partial. |
| 2. There is pain in back, extending down the legs and around the body. | 2. No fixed pain in back or legs, and no "band" around the body. |
| 3. Passing ice or hot water down spine gives great pain. | 3. None is experienced. |
| 4. Jerking or spasm of muscles of legs exists. | 4. None. |
| 5. Bladder and rectum are affected. | 5. Not so. |
| 6. Prickling and formication occur. | 6. None. |
| 7. As time passes the paralysis becomes worse. | 7. As time passes it diminishes. |
| 8. There is great tendency to bed-sores. | 8. None whatever. |
| 9. Extremities are colder than normal. | 9. Temperature normal. |
| 10. Sensibility impaired. | 10. Not so to any great degree. |
| 11. Muscular enfeeblement is strictly confined to the lower limbs. | 11. There is often a tendency to enfeeblement of upper limbs. |

Dr. G. remarks that in the treatment of organic paraplegia "the indication is to disgorge as much as possible the vessels of the cord, and produce a sedative, quieting effect upon it. In the functional variety the reverse is true; we should endeavor to stimulate the functions and circulation of the cord."

It would appear from the former part of this statement that Dr. G. considered all organic affections of the cord as essentially inflammatory, and as requiring antiphlogistic local and general treatment. The causes of functional paraplegia, generally, he notes as follows:

1. Blood poisoning, *e. g.:* Syphilis, Lead, Arsenic, Diphtheria.
2. Intense irritation of any part of the nervous system, *e.g.:* Worms, Dentition, Disease of genito-urinary system, and any acute affection.
3. Hysteria.
4. Great impoverishment of the blood, as after malarious fevers, parturition, etc., etc.

To the conjoint statements, we beg leave to "plead the general issue" that organic diseases of the cord resulting in paraplegia are by no means necessarily of an inflammatory character requiring means to disgorge its vessels and produce a sedative quieting effect upon it; there are frequently cases where, even though organic disease be present, all those agencies which the author would restrict to "functional derangement, become immediately useful. We object *in toto* to the term "functional" in this connection. Perhaps Dr. S.'s idea was to diagnose the so-called inflammatory affections of the cord from those where its structure does not manifest those gross and striking lesions generally referred to inflammation. We suggest that: That usually denominated *inflammation* in any part is a general term embracing a number of varieties of modification of nutrition of that part.

All those agencies which he characterizes as productive of "functional" paraplegia, likewise disturb the nutrition of the cord, either directly through the blood, or through the nervous system, in either case simply modifying the nutrition—

changing the molecular structure of the part—and—consequently *the lesion is always organic.*

The division given is a useful one insofar as it goes to counteract the baleful antiphlogistic treatment, too often indiscriminately applied to all cases of paraplegia.

---

*Rest and Nature.*—Mr. Hilton concluded his invaluable series of lectures on "The Influence of Physiological and Mechanical Rest in the Treatment of Surgical Diseases and of Accident," delivered in the theatre of the Royal College of Surgeons, with the following words:

"I have always thought, with some others, that disease is nothing more than an abnormal arrangement of, or a simple deviation from, normal conditions; and that physiology should be made the basis of pathology, and of the deductions therefrom essential to the guidance of accurate surgical practice. And it struck me that, in discussing the question of the influence of rest in the treatment of disease generally, I might, more especially by pointing out its application to the disease of the joints, induce in the minds of some of our professional brethren a more philosophic consideration of the pathology and treatment of a class of cases which are not unfrequently abandoned to the care of the empiric and the mindless.

By regarding this subject of physiological and mechanical rest in what I conceive to be its proper professional light, the surgeon will not only discover and readily interpret the exigencies and necessities of man's most wonderful, most comprehensive, and most perfect mechanism, but he will be compelled to admit that he has *no power to repair directly* any injury which this machinery may have suffered, or to supply the minutest living atom towards filling up the gap made by disease. It will induce him to acknowledge, in all humility, that it is the prerogative of vitality to repair the waste of its subservient structures, and to realize that the surgeon's chief duty consists in ascertaining and removing those impediments which obstruct the reparative process or thwart the efforts of Nature to redress injury, and thus enable her to revert to her normal existence."

*Mutatis mutandis*, the same words are applicable in the practice of medicine as well as surgery—the idea of which is gradually getting more and more clearly defined in the professional mind. That physician whose whole stock in trade consists merely of a list of symptoms for which certain medicines are to be used, is not up to the level of the times, whether he give calomel or infinitesimals. "It is the prerogative of vitality," by which we understand the organizing processes within the depths of the organization itself,—to perform the cure. And yet be it observed, that this does not detract a hair's breadth from the importance of the physician's office—it does not, as some absurdly suppose, leave everything to nature. Far from it. "Nature only cures," says Cabanis, "in peculiar circumstances and under certain conditions—whereas by art we are enabled to change the former and to fulfill the latter." Thus this

————"is an art
Which does mend nature,—change it rather; but
The art itself is nature."

"It is the really enlightened physician alone," says Sir Gilbert Blane, "who can discern in each particular case, to what extent art is availing, or if it is at all availing. * * * Those who conceive the whole art of medicine to consist in wielding the powers of the Materia Medica, entertain a narrow and unworthy view of their own duty and of the value and dignity of their profession."

True enough is what Hippocrates says: *Natura sibi invenit vias, et inerudita existens, quæ expediunt perficit*—but it is just as true that the ignorant man is a thousand times further from nature than he who is wise. It is the object of true wisdom to correct the mistakes which ignorance makes with regard to nature. Positively to unlearn the errors of the eyes and ears, which constantly deceive the uninstructed. And this to the medical man is especially important. For, as Blane writes, "from all I have been able to observe, the ignorant person is more apt to counteract nature by pernicious interference, than the wildest professional theorist; nay, ten to one, he is the greatest theorist of the two, for every old woman has her theory generally drawn from the humoral pathology."

Which also is worth thinking of!

Some writer a few years ago hit the point exactly thus: "The acquirement of our professional learning requires a *scholar;* its practice requires an *artist;* its standing and social relations demand a *gentleman.*" Is that a trinitarian doctrine too mysterious to be comprehended? We trust not.

---

*Cancer Treated with Arsenical Mucilage.*—Dr. W. Marsden, Surgeon to the Royal Free and Cancer Hospitals, reports four cases of cancer cured by local application of arsenical mucilage. He states that the application was rigorously limited to the fungous structure, experience having told him that its application to the healthy tissue is fraught with extreme danger. The proportion of arsenic is not given. It seems to be pretty well established, that the stronger the local application, the less danger is there of absorption to the general system.

---

*Inter Orbitar Aneurism.*—M. Ernest Hart reports a case of Intra Orbitar Aneurism cured by ligature of the carotid after failure of digital pressure. Of twenty cases of this affection on record, treated by ligature of the primitive carotid, thirteen were completely successful; three incompletely so; three died, and one was wholly unsuccessful.

From the success of Brainard (in a case where the ligature failed) with an injection of a solution of lactate of iron (eight grs. to the ounce), and M. Bourquet's case, when he used an injection of solution of perchloride of iron, he observes that the treatment by injecting coagulating fluids is worthy of being recorded among our surgical resources, as often preferable to the ligature. Digital pressure is to be preferred where it can be tried. This has proved successful in eighteen days, the total duration of the pressure being only seven hours and twenty minutes.

---

*Ovariotomy—Death by Tetanus.*—Dr. E. S. Cooper recently performed ovariotomy. The patient passed on admirably until the eighth day, when, by domestic neglect or brutality, she was exposed to a severe draught of cold air. A severe chill soon ensued, followed by tetanus, under which she sunk upon the ninth day.

*Amputation of the Fore-Arm.*—The Lancet states that excellent stumps are secured by a modification in the usual process of amputation of the fore-arm, at St. George's Hospital. "It consists simply in making circular incisions through the skin of the fore-arm, drawing it well back, then making flaps of the muscles, and finally sawing through the bones. The healing process goes on with great rapidity, and the ends of bone have a firm substantial cushion of muscle which is perfectly covered by the skin. There is no redundancy of material, but a smooth, even, comfortable feeling stump," well adapted for affixing an artificial hand.

*An Old Case.—Serous Cyst Connected with the Kidney.*—Richard B., aet. 20, late from England. Eleven weeks before had nephritis, treated by vigorous antiphlogistics. Previously healthy. Did not entirely recover, but able to go about. Exposure to cold, etc., put him to bed again with evidences of ascites. The attendants "drenched him" with Cal., Gamboge, Colocynth, etc. Paracentesis *at the navel.* One side only was emptied, the other remaining tumid and tense, with distinct fluctuation. This side was punctured, *at a venture*, and about a gallon of serous fluid discharged. General anasarca and general debility both being present, and *both* sides filling up again, among others the writer was called in council.

One Dr. S. supposed that the side which refused evacuation through the navel, had an accumulation of fluid between the peritoneum and the abdominal parietes. Another Dr. S. said it was clearly an abscess in the cellular tissue of said parietes. Another Dr. present thought it could not be ovarian dropsy because the patient was a man, but what else it might be he had not the remotest conception. Dr. H. thought it was enlargement of the spleen, but, being in altogether a different part of the abdomen, he supposed there must be an abnormal arrangement of the viscera—besides this he concluded there was disease of the liver and mesenteric glands. On the whole all but one of the party concluded the liver to be "at the bottom of it." No matter what the writer said or thought, it was not regarded. Salts and Senna were given, as Calomel had been in large doses. The cyst and abdomen both filled rapidly. Calomel and Squills were given every two hours; Mur. Tinct. Ferri every eight hours. Another hole was cut in the side, the water drawn off and a long catheter introduced on a tour of exploration. Another hole was made at the umbilicus to draw off the fluid from the peritoneal cavity. In twelve hours the distension was as great as ever. A string of candle wicking was then threaded in the eye of the catheter, and passed from one aperture to the other, and left there. The fluid drained off at its leisure. The distension was overcome, but unfortunately the patient died some eight or ten hours after.

*Post mortem* revealed a large serous cyst attached near the lower extremity of the right kidney, that part of the kidney being completely disorganized. The cyst would hold about six quarts, the walls being made up in considerable part of the mesentery, mesocolon and momentum—a perfect riddle of morbid anatomy; the original sack seeming to have been developed by sheer distension of the peritoneal covering of the kidney. Its contents had no urinous odor. The other viscera of the abdomen were healthy. Examination of the thorax was not permitted.

There are no further notes of the case—this fragment only having floated away scorched by the flames which years ago burned up the notes, comments and reflections upon this, and a multitude of other cases, which the writer wrote out in the earlier years of practice.

The writer and one other of that original "Council," are now the only survivors, for that was many years ago. The other survivor will never see this sketch of that old consultation—for it was twenty years before that time, (as never since,) that he had seen a medical book, much less a medical journal.

---

*An Astounding Case.*—Not many years ago, and not a thousand miles from this city, occurred a death under circumstances indicating upon the part of the patient the most sublime disregard of the "authorities."

The case was in charge of a doctor, long celebrated for his unflinching faith in Hydrarg. Subchlorid. The patient was purged therewith, and therewith again and again purged. "Alterative" doses were given, interspersed with occasional purgings. But down he went, to the utter astonishment of the Doctor, who is nothing if not "heroic" with calomel. In spite of purging and salivation, the patient died. Believing the case extraordinary in the extreme, in connection with several professional gentlemen, he made a *post mortem.* Nothing was found by the scalpel to account for the death. Dropping his dissecting knife under the profound agitation of his feelings, the Doctor exclaimed, whilst his spectacles shook with his emotions: "The secretions were perfectly restored—half an hour before he died he had a regular *spinach* stool. I wonder what in h—l he died of!"

It may be observed that the public saw nothing especially remarkable in that case—any more than it did when the Banker died. The public had become accustomed to that kind of result. Every day wonders do not astonish us.

---

*Spina Bifida Cured by the Iodine Injection.*—Dr. Lezerie reports, in the *Moniteur des Sciences Médicales*, Oct. 8th, 1861, a case of spina bifida in an infant of 9½ months. The tumor was in the lumbar region, and as large as a moderate sized orange. Paralysis of the lower limbs, and partial paralysis of the upper, but no hydrocephalus. It had twice spontaneously opened and then refilled. M. Lezerie operated in the usual manner and with the proper precautions. Two injections of equal parts of Tinct. Iodin. and water were made, having first allowed part of the contents to escape. During the progress of the case, compresses dipped in an iodine solution were kept over the tumor. The success was marked, the paralysis commencing to disappear in a few days. Twenty-seven months after the operation, the tumor was found reduced to the size of a hickory nut, the child well developed, running about and with no trace of her former condition, save incontinence of urine. A renewal of the operation at an early period would probably have abbreviated the duration of the case.

---

*Why Was It?*—An army surgeon, from Wisconsin, who has been with his regiment in Tennessee, writes us with regard to the statement, made in the June number of this JOURNAL, (p. 348,) about the success of a volunteer surgeon in reducing the sick list of the regiment to which he was detailed from 349 to 80 in a single week. He says: "Now I would like to know why it is he could succeed with astringents, while dozens of good surgeons could do nothing with the disease (camp diarrhœa) under this treatment? I think his treatment calculated to do harm in every case of camp diarrhœa where it is applied."

Our friend will recollect that we only "did tell the tale as it was told us," and called upon the actor-in-chief to give us the facts over his own signature. To this call there has been no response. Our own private opinion is that the Dr. made the astringent decoction with the addition of Capsicum and Carb. Ammonia, *exceeding mild*, and cultivated the hospital cook and good regimen, whereupon the decoction (said to have been made by the barrel,) became quite tolerable and readily to be endured. We need not repeat for the hundredth time that we have no faith in specifics, or specific mixtures, for this "plague of the armies."

Our correspondent continues: "I think the disease consists in, or is caused by, congestion of the portal system, irritation and many times inflammation of the mucous coat of the large intestine, vitiated secretions, and, in many cases, paralysis of the lacteals and exhalants, so that the one does not take up the chyle, and the other pours out the watery constituents of the blood in exhausting quantities, indicating in my opinion, the implication of the ganglionic system.

"Now if this state of affairs exists, can it be that a course of astringents will be of any permanent benefit? If so it is different from any of your teachings that I have ever listened to, and certainly different from my own experience."

We beg our friend's pardon for extracting thus much from his private letter, but as it hits several nails precisely on the head he will certainly forgive us. The causes of camp diarrhœa are as multifarious as the causes of diarrhœa in civil life, and precisely the same principles of treatment are applicable with chances of success in equal ratio provided its causes are withdrawn. There is no Morrison's Pill about the matter.

---

*Glycerole of Aconitia.*—The *Druggists' Circular* replies to an enquiry by a Chicago correspondent that the Glycerole of Aconitia "is probably the best mode of use of the alkaloid. It is prepared by adding to the powder enough acetic acid to dissolve, and mixing with glycerine. No standard strength is given; one grain per drachm of glycerine would most likely be best."

---

*Lapis Vulnerarius.*—The *Circular* gives this formulary: ℞ Aluminis ℥ iij; Zinci Sulphat. ℥ iij; Cupri Subacet. gr. iv; Ammon. Chloridi, gr. iv. Fuse together in a crucible and add two grains of powdered saffron.

---

*Cleaning Bones.*—The same paper directs for this purpose a pound of Chloride of Lime to two gallons of water, and add a solution of about an ounce of bicarbonate of potassa, cold.

*Administration of Cod Liver Oil.*—M. Dannecy, in the *Union Medicale* recommends the exhibition of eight or ten grains of magnesia suspended in water after each dose of the oil. It is said to be completely successful in preventing the vomiting so apt to occur even some hours after taking the oil.

---

*Cement.*—Six parts of plumbago, three of slacked lime, eight of sulphate of baryta, and three of boiled linseed oil makes a very superior cement for laboratory use, as also for gas or water tubing, steam pipes, boilers, etc.

---

*Antiseptic Solution.*—M. Leprieur commends arseniuretted alcohol for the preservation of objects in natural history. They should be immersed in the solution twelve hours and then dried, when they will no longer be subject to the destructive attack of insects, larvae, etc., as well as preventing their own decomposition. No other means has been found as efficacious.

---

*Tartarized Antimony Pustulation of Nævi.*—Strickland Kingston, M. D., in the *London Lancet*, recommends that the base of the nævus should be freely transfixed and punctured in different directions by the acupuncture needle, and then covered to the thickness of a sixteenth of an inch with an ointment composed of from two to four grs. of Tartar Emetic to eight grs. of Spermaceti. The scabs of the subsequent pustules are allowed to drop off spontaneously when the nævus will be found healed.

---

*Embalming.*—The body of an individual having very peculiar development, the peculiarities of which were that a form of perfect symmetry was surmounted by a head the fac-simile of that of the gorilla, the face covered with coal black hair, yet belonging originally to an "intelligent, kind and accomplished" female, is preserved and on exhibition at No. 191 Piccadilly, London. She died five days after giving birth to a child, the fruit of legitimate wedlock. The child's body is also preserved by the same process. The bodies stand before the observer "without the slightest odor, the slightest change or slightest appearance of corruption." The *London Lancet* is promised the particulars of the process by which these bodies have been thus preserved unchanged for a long period.

If published generally it is to be feared that Mr. Artemas's "wax figgers" will speedily be thrown into the back ground.

---

*Hallucinations of Chloroform.*—The *London Lancet* records another case in which a female accused a dentist of having criminally assaulted her whilst under the influence of Chloroform. The charge was dismissed by the magistrate as unproven, and the dentist brings a cross action for defamation of character. Meanwhile it is well to recollect, that while no man can prophecy what a woman will say or do when not under Chloroform, the uncertainty is materially increased when she is. That potent agent, therefore, should never be administered unless in the presence of other parties, and, if possible, a female friend of the patient.

---

*Bread-Making by Machinery.*—This process which has so long been in vogue in this country that it has ceased to attract public attention or comment, is just beginning to get itself talked about "over the water." We expect to hear by the next steamer that an injunction has been sued out against it, as in the instance of our enterprising, and somewhat talkative countryman, George Train's street railroads. The *Lancet*, however, thus far pats the project on the back.

---

*Vital Electricity.*—*Galignani* has a "sensation" in regard to recent experiments, going to show that the living frame has a direct action on the magnetic needle,—"what consequences may be evolved therefrom, time alone can show."

---

*About Mercury and Blisters.*—At the outset of various

"inflammatory" diseases, mercury, in cathartic doses, is undoubtedly antiphlogistic. By its evacuant action it causes the bile, a liquid rich in carbonaceous, and consequently calorifacient, matters, to escape absorption and pass out of the system. In this regard, under certain conditions, well understood, it is one of the most efficient cathartics. From this point, until the exuded material of inflammation becomes consolidated, there is no warrant for its use. When the exudation consolidates in its new situation, there is reason to believe that it may become useful again, by its power of stimulating or accelerating the molecular change which must occur before the new formation can be reabsorbed. But when the molecular change passes to suppuration, the mercurial again becomes unwarrantable.

Practically, the time for the administration of mercurials, in "alterative" doses, corresponds with, or is subsequent to, the "blistering point," so clearly designated in all our best standard treatises on practice.

The facts in our possession warrant the assertion that employed before this point is reached, alterative doses of mercury are worse than useless.

Long lists of recoveries can be given where "alteratives" have been relied upon from the very outset of the disease, but it is now safe to remark that the beneficial results attained depended wholly upon, first—the primary cathartic action of the Mercurial, and, second, upon the opiate with which, from time immemorial, the "alterative" has been combined.

The "alterative" dose of mercurial is not antiphlogistic, but *stimulant.*

Slowly the best observers have reached this truth, particularly having been obliged to recognize it in hepatitis, and speedily after in other phlogoses.

The result is, the range of application of mercurials has been amazingly restricted, but, under the appropriate conditions, resort to them is had with immensely greater confidence and certainty.

There is something of a similar history to blisters. Formerly in every case of inflammation the blister was clapped on externally at once, and the Calomel exhibited internally at the same moment. Hence the measureless discussions about their utility—until the happy idea of the blistering point struck shrewd and cautious observers, since which time they have been largely restricted in application.

Nevertheless their so frequent use in "inflammatory" affection fostered the notion of their being "antiphlogistic" in their effects. Yet practical men have again and again resorted to them for their *stimulant* impression. The simple fact is the blister is not "antiphlogistic," but *stimulant*, in precisely the same sense that mercury is stimulant, that is it hastens molecular change, not only in the part of application but also in the part which it is in physiological relation, either through the nervous ("reflex") system or by the circulation.

In this sense the application of a blister to the thorax in pneumonia, has an internal influence altogether identical with that of a stimulant applied to an external inflamed part—(the conjunctiva for example)—and with similar danger of error in application, dependent on the exact condition of the part.

Practically there is no therapeutic fact better established than that, to favorably impress certain internal inflammations, particular external parts are to be selected for applying of the blister—a phenomenon which utterly disproves the idea which has been frequently advanced by enthusiastic admirers of Cantharides, that the medicine acts only through the general circulation. The effect cannot be imitated by exhibition of the cantharides internally, neither by their application externally at other parts. Pneumonia requires the blistering cerate over the part, and will not be benefited by vesicating the arm or the leg—yet the arm is anatomically the nearest, and the leg the more remote, so that, in the latter case, the old theory of *revellent* action fails, as in the former does the circulatory hypothesis.

More inquiry is requisite in each case as to the exact kind of modification of nutrition which is present, that under the general and vague term "inflammation" may not be confounded (as is now too frequently done) a great variety of morbid conditions.

Meanwhile it is to be recollected that healthy blood is the best modifier of nutrition, the best alterative, the best resolvent. The practical induction is obvious.

---

*Local Bloodletting.*—Since the striking discontinuance of the heroic general bloodletting, found so useful in the diathesis of fifty and a hundred years since, frequent resort is had to local abstraction of the fluid by leeches, cups and incisions. This seems to have been adopted as a "compromise measure," and some seem to think it positively a "constitutional" necessity.

Thus it is recommended in typhic diseases where symptoms of local disorder exist; in affections of all sorts where, under the diagnosis, the nomenclature demands the termination *itis*, and yet the systemic condition is such as positively to forbid general venesection, or even medicinal arterial sedatives; and in a multiplicity of chronic local derangements, where other measures fail.

We think we express a general truth when we say that in typhic diseases, local bloodletting is to the full as objectionable in principle and practice as general would be. Yet the molecular changes at the part of application may prove useful, precisely as the blister proves useful, by its stimulant effect upon the organ with which it is in physiological relation—the abstraction of blood is an injurious attendant of the method, and both physiology and experience demand its discontinuance.

A large amount of blood, especially in children and adults of a hæmorrhagic diathesis, may be drawn by leeches, and then the general effect is exactly that of the old venesection. The shuddering disgust with which children and nervous adults look upon the wriggling, slimy, snaky leech, may produce a sedation—but it scarcely seems necessary to adopt this method whilst better ones are at hand., The cupping apparatus, the scarificator and the knife respectively may produce a strong mental impression—and so did "the rope with which a man was hanged," "the powder of a human skull," and "the parings of an ass's right fore-foot," but now-a-days the latter are not frequently employed.

It is about time that this irrational and unphysiological practice should be dropped from the text-books, as it has pretty much disappeared from the actual employment of our best practitioners. The local action is better secured by those well-known methods—cold, positive or by evaporants; sedative or anodyne lotions, etc.; heat by dry or moist appliances; stimulation by rubefacients, vesicants or suppurants—the dry cup, etc., etc. Local medication possibly may exercise an "alterative" influence. Practically—in nine cases out of ten where the local bloodletting is (or rather *was*) directed, both general and local stimulation are demanded. But, *above all*, wash the diseased organ with healthy blood *from within.*

---

*The Army Surgeon Again.*—In reply to the somewhat damaging comments of one of our correspondents, our friend Prof. H. A. Johnson sends us the following "letter of credit" for the benefit of the aggrieved individual. It does credit to the kindly heart of our genial friend. The benevolence of feeling which prompts it is really refrèshing in these cold-blooded times, when self-sacrificing acts of disinterested friendship are so exceedingly rare. Our sympathy with its philanthropic intent causes us to insert it most cheerfully.

[For the Chicago Medical Journal.]

Messrs. Editors:—

In your May number I find an article headed "*Pittsburgh Landing*" and signed "R." in which Brigade Surgeon Robert Roskoton is made the snbject of severe criticism. I know nothing of the facts there alluded to, but I do know from an intimate personal acquaintance, with the very best opportunities of judging, that Dr. Roskoton is a highly intelligent and cultivated gentleman, and that his attainments as a physician and surgeon are second to few, if any, in our country. He is a German, it is true, but he is an educated, thinking man, and a fitting representative of that class of German physicians to whose patient philosophical researches our profession is so largely indebted.

Very truly, H. A. JOHNSON, M. D.

---

*A Practical Treatise on the Diseases of the Heart and Great Vessels, Including the Principles of Physical Diagnosis.*—By WALTEN HAYLE WALSHE, M. D., F. R. C. P. Prof., etc., etc. A new American from the Third Revised and much enlarged London Edition, Philadelphia, Blanchard & Lea, 1862. pp. 420. From the publishers.

The late period in the month at which this work was received, must preclude an extended notice of it in this No. of the JOURNAL.

Fortunately, the established character of the treatise, and the world wide reputation of the author, render it scarcely necessary to do more than announce the issue of a new revised and much enlarged edition. It is a standard and truly classic work. We are sure that we do injustice to no other author when we say that it deservedly stands at the head of all authorities upon the subject. To the medical student it is invaluable for its lucid and graphic descriptions; to the medical teacher for its scientific accuracy and comprehensiveness; to the general practitioner for its masterly diagnosis, and its clear and sagacious directions of treatment. In a subsequent No. we shall find occasion to glance at the contents of the book more in detail.

---

*Braithwaite's Retrospect of Practical Medicine and Surgery. Part the Forty-Fifth. W. A. Townsend*, 39 *Walker St., N. Y.* $2.00 a year in advance. Half-yearly parts $1.25—Postage paid by the Publisher.

This number of the well-known Retrospect is fully up to, and in some respects surpasses, its predecessors. As a *resume* of foreign advances and practice it is unsurpassed.

*A Practical Guide to the Study of Diseases of the Eye; their Medical and Surgical Treatment.* By HENRY N. WILLIAMS, M. D., Fellow of the Mass. Med. Society, etc., etc. Boston: Ticknor & Fields. 1862.

A book from this well-known and distinguished professional gentleman cannot fail to be both valuable and popular. Our copy was placed in the hands of a medical friend for review, and as he has not yet favored us with his comments we must waive further notice until next month.

---

*Miscellaneous Items.*—The numerous friends of Brigade Surgeon J. V. Z. BLANEY, will be gratified to hear that he is still continued at his post in this city, the order detaching him to report at Washington having been revoked....Brigade Surgeon CHAS. L. ALLEN, Prof. of Theory and Practice in the University of Vermont, is ordered to report to the Surgeon General as a member of the Board for the examination of Surgeons of Volunteers....Assistant-Surgeon General Wood is to act as Surgeon General of the Western Department, with head-quarters in this city. The peculiarly favorable situation of Chicago making it pre-eminently the medical centre of the North-West....The State Examining Board are now in session and, we understand, are discharging their laborious duties with zeal and fidelity....Dr. L. D. Boone, an old resident and distinguished practitioner of this city, has been put under arrest by the military authorities for, it is alleged, some infraction of the regulations of Camp Douglas. We trust that his detention will be but brief, unless, as we cannot believe, the offence is more serious than we have understood. No class of the community has given evidence of more fervid patriotism than the physicians....A recent case of ovariotomy in this city proved completely successful. We hope to give the particulars in an early issue....A case of rupture of the uterus, escape of the child and secundines into the cavity of the abdomen, the large abdominal section after the lapse of several hours, with a hopeful condition of the woman for several days, but ultimate death from pyræmia, has recently attracted attention in the city. Details will probably be published soon.... Physicians, dentists and others who have been wheedled into buying a liquid preparation to facilitate soldering, of an enterprising vender, might have saved their quarters by recollecting that a simple solution of chloride of zinc entirely answers the purpose—the melted solder diffuses itself rapidly over the surface moistened with it....A few grains of perchloride of iron introduced by the side of an ingrowing toe-nail will, generally, so condense the tissue, that it can in a few days be readily removed, leaving a healthy surface—so says Dr. Miner in corroboration of others....There is a great demand for Staff Surgeons in the army, but it is said that, of a large number who have been examined, very few have been found thoroughly qualified. The reason is obvious—medical men of high standing and large experience do not feel like subjecting themselves to a school boy examination or the petty insolence of youthful examiners—even for the ungentlemanly pittance and position set apart for them by the laws. They had rather serve as private soldiers and carry a musket and knapsack...Dr. Horace Green, of New York City, has offered $20 to each of the first fifty volunteers in the county of Westchester, N. Y. This argues splendid profits from the throat cauterizing specialty.... Our friend, Dr. Holmes, whose first foreign letter appears in this No. of the Journal, neglects to chronicle one of the most interesting personal items, which we ourselves scarcely dare more than to breathe, to wit: that he has taken captive, without fear of extradition treaties, one of Vienna's fairest and most accomplished daughters. It is not believed, by those most intimately acquainted with the Dr., that the prisoner will ever wish an exchange, but will ever make glad one of our American *Holmes*....Under the new law reorganizing the Med. Dep. U. S. A., the appointment of Medical Inspector General has been conferred on Thomas F. Perley, M. D....Dr. Ellerslie Wallace was recently elected Prof. of Obstetrics and Diseases of Women and Children in the Jefferson Medical College.... Dr. Charles Clay, of Manchester, advocates in the Glasgow *Medical Journal*, in cases of *placenta prævia* simple detachment of the placenta from the os by the forefinger, leaving the rest to nature. According to our experience this is by far the safest, as it is thesimplest method of treatment. Statistics are immensely in its favor....Prof. C. A. Lee, writes that the London Surgeons operate more fearlessly, and with greater rapidity than Americans, but he very much doubts whether as successfully, except in particular cases.

